# Prevalence of child and adolescent psychiatric disorders in India: a systematic review and meta-analysis

**DOI:** 10.1186/1753-2000-8-22

**Published:** 2014-07-21

**Authors:** Savita Malhotra, Bichitra Nanda Patra

**Affiliations:** 1 Department of Psychiatry, Postgraduate Institute of Medical Education & Research, Chandigarh 160012, India

**Keywords:** Epidemiology, Weighted prevalence, Psychiatric disorders, Meta-analysis, Child and adolescent

## Abstract

**Background:**

The importance of epidemiological studies lies in recognition of cases that do not come to treatment settings. The increasing focus on child adolescent mental health in India points to the necessity of epidemiological studies on children. Although there are a few such studies done in different parts of India in different socio-cultural settings, data from those cannot be generalized to the entire country. This need can be served by meta-analysis. There has been no meta-analysis reported from India for the child and adolescent psychiatric epidemiology.

**Aim:**

To review and do the meta-analysis of epidemiological studies on child and adolescent psychiatric disorder from India.

**Methods:**

Sixteen community based studies on 14594 children and adolescents; and seven school based studies on 5687 children and adolescents, reporting prevalence of child and adolescent psychiatric disorder were analyzed and overall prevalence was calculated.

**Results:**

The prevalence rate of child and adolescent psychiatric disorders in the community has been found to be 6.46% (95% confidence interval 6.08% - 6.88%) and in the school it has been found to be 23.33% (95% confidence interval 22.25% - 24.45%).

**Conclusions:**

This is the first meta-analysis determining the epidemiology of child and adolescent psychiatric disorders in India. It has been found that the reporting systems of psychiatric disorders in children are inadequate.

## Introduction

Psychiatric epidemiology is the study of the distribution and determinants of occurrence of mental illness in human beings. In India many investigators have studied the prevalence of various psychiatric disorders over period of time. In a country like India where people are less aware about mental health problems, only patients with major mental illness access care and those with minor mental disorders remain in the community without identification and management [1]. So the advantages of the epidemiological studies lie at targeting all levels of recognition of the minor cases; missing cases; and of the new cases.

In India, the total number of children in the age group 0-6 years as per the population totals of Census, 2011, is 158.8 million, which is 13.12% of the total population [2]. The percentage of population in 0–14 year age group consists 30.9% of total population of the country [3]. In India, the adolescent population constitutes a quarter of the country’s population which is approximately 243 million which in turn constituted 20% of the world’s 1.2 billion adolescents [4]. Children and adolescents in low and middle income countries (LAMIC) constitute 35–50% of the population [5]. About half of all lifetime mental disorders begin before the age of 14 years [6,7]. As per a review by Sharan & Sagar [8], worldwide prevalence rates for child and adolescent mental disorders are around 10% -20% [9]. There have been gap between needs and services for mental health, especially in low and middle income countries (LAMIC). Most care is institutionally based with poor attention to community mental health [10]. Psychiatric epidemiological studies from high-income countries indicate that more than a quarter of children and adolescents meet lifetime criteria for a mental disorder [11]. The differences in sociocultural factors and health systems probably limit the generalisability of research evidence from developed countries to low-income and middle-income countries [12]. The evidence base on the burden of child and adolescent mental disorders in LAMIC is relatively small due to a number of factors: in particular, insufficient skilled human resources, low awareness and low priority, high service load, greater concern for child mortality than morbidity, and journal acceptance biases against LAMIC research. However, some epidemiological evidence is now available from a number of LAMIC which shows the existence of mental and developmental disorders, and the demonstration of their impact on health care seeking and other aspects of the lives of children and adolescents [5]. Previous epidemiological studies have found that the prevalence of child and adolescent mental disorders to be 17.7% in Ethiopia [13], 15% in Bangladesh [14], 12.7% and 7% in urban and rural Brazilian school sample respectively [15,16] and 6.9% in Puerto Rico [17].

In India, though many investigators have studied the prevalence of psychiatric disorders in children, and there is a wide variation in prevalence rate from 0.48% [18] to 29.40% [19]. Apart from this, there were also some other limitations of the studies like use of non-representative samples and small population [20], unspecified or non-standard criteria for diagnosis and classification [21] and high dropout rate after screening [22]. And also the absence of empirical data on the magnitude, course, and treatment patterns of mental disorders in a nationally representative sample of children and adolescents has impeded efforts essential for establishing mental health policy for this population. Research initiatives should address the lack of national statistics on mental health in children [23]. By looking at the results of any one study, it is difficult to obtain firm estimate of the overall prevalence and extrapolation of results of one study to the whole population becomes questionable, particularly in a large and culturally diverse country like India. Some of these issues can be addressed by meta-analysis, in which finding from a number of studies can be pooled to get the overall estimate.

There are two meta-analytic studies reported from India on the prevalence of psychiatric disorders in adults [24,25], but no meta-analysis has been reported for the child and adolescent psychiatric epidemiology. Only one review has been reported for the child and adolescent psychiatric epidemiology in India, which was more than a decade ago [26]. As meta-analysis involves systematic analysis and synthesis of results from several studies done on a subject, it was felt that an attempt should be made to combine the results of various epidemiological studies done so far to calculate the overall and the best estimate of prevalence of psychiatric disorders in children and adolescent in India.

### Selection of studies

Extensive search was done on Pubmed, Indian Journal of Psychiatry, google search for published psychiatric epidemiological studies done in India on child and adolescent population using keywords “Psychiatric disorders”, “Prevalence”, “Community”, “Child and adolescent”, and “Epidemiology”. Cross references of the psychiatric prevalence studies and extensive manual search of different journal was also done. All the studies included in the meta-analysis have been done on representatives and probabilistic samples to enhance the value of meta-analysis.

Studies were selected as per the following criteria.

1. Studies carried out in India in the community or in school. The community based study and the school based studies were analyzed separately because the prevalence of mental disorders are differently presented in the two settings.

2. Availability of separate prevalence reports for rural and urban areas in case of mixed studies. This was done because the childhood psychiatric disorders may depend upon socioeconomic status and urbanisation. In rural area there may be greater social stability and support which may act as a protective factor [5].

3. Coverage of child and adolescent age groups up to the age of 19 years (as per the WHO guidelines) with the exception of two studies which examined the prevalence among child and adolescent up to 20 year age, but had sound methodology and large sample size.

4. Children and adolescents should have been clinically examined by the investigators. This is because the diagnosis made after evaluation by expert clinician using all the available data is more valid [27].

5. Studies on specific disorders [8,28-36], unpublished studies; and studies done on high risk groups [37-43] were not included in this analysis.

6. Study estimating the period prevalence rates [20] was also not included.

A total of 28 community based studies were found. Out of 28 community based studies one study by Dube et al. [44] was excluded as per criteria-2, another one by Hacket et al. [45] was excluded as per criteria-4 and 3 studies were excluded as they were done on specific disorders [8,28,29] and another 7 studies were excluded as they were done on high risk groups [37-43]. One study by Lal and Sethi [21] was excluded as per criteria 6. Hence sixteen community based studies fulfilled the above inclusion and exclusion criteria and were included in meta-analysis and are shown in the Table 1.

**Table 1:** Community based studies included in analysis

**Sl. No. P.I.**	**Year**	**State/UT**	**Setting**	**Age group**	**N**	**Prevalence (%)**	**Instrument/Diagnostic system used**
1. Sethi [46]	1967	UP	urban	0-20	924	7.68	Comprehensive questionnaire, Clinical interview
2. Elnager [48]	1971	WB	rural	0-14	635	1.3	Clinical interview, WHO expert committee on mental health (1960)
3. Sethi [47]	1972	UP	rural	0-20	1386	5.84	Comprehensive questionnaire, Clinical interview
4. Verghese [57]	1974	TN	urban	4-12	747	8.17	pre-tested questionnaire, validated mental health item-sheet, ICD-9
5. Nandi [49]	1975	WB	rural	0-11	462	2.6	questionnaire, WHO expert committee on mental health (1960)
6. Thacore [51]	1975	UP	urban	0-15	1191	6.9	Clinical interview, DSM II
7. Shah [52]	1980	Gujrat	urban	4-14	1089	0.8	Symptom checklist, Clinical interview
8. Singh [19]	1983	UP	urban	1-14	279	29.40	Clinical interview, WHO expert committee on mental health (1960)
9. Nandi [18]	1986	WB	rural	0-11	551	1.08	questionnaire, WHO expert committee on mental health (1960)
10. Mehta [53]	1985	TN	rural	0-14	2012	1.84	Indian psychiatric survey schedule, clinical interview
11. Sachdeva [55]	1986	Punjab	rural	0-14	660	1.06	Indian psychiatric survey schedule, clinical interview, ICD-9.
12. Nandi [50]	2000	WB	rural	0-11	1173	2.73	questionnaire, WHO expert committee on mental health (1960)
13. Singh [56]	1989	Rajasthan	urban	5-15	348	14.37	questionnaire, clinical assessment
14. Premarajan [58]	1993	Pondy	urban	0-12	273	5.86	pre-tested questionnaire, validated mental health item-sheet, ICD-9
15. Gaur [59]	2003	Haryana	mixed	6-14	800	16.5	Diagnostic Interview Schedule for Children, ICD-10, childhood psychopathology measurement schedule (CPMS)
16. Srinath [61]	2005	Karnataka	mixed	0-16	2064	12.5	screening checklist, child behaviour checklist, additional module, children’s behaviour questionnaire, felt treatment need, diagnostic interview for children, structured interview schedule, parent interview schedule, Vineland social maturity scale (VSMS), Binet Kamat test, SLD battery, and children’s global assessment scale. Diagnosis was made as per ICD-10.

### Methodologies of included community based studies

There are several difficulties in defining clinical cases in epidemiological studies. The presence of disorder implies clinical significance or need for treatment. Lay interviewers do not have experience to judge clinical significance, so likelihood of missing out common mental disorders during screening process is high. In view of the common methodological limitations, methodologies adopted in the studies included in this analysis (Table 1) were evaluated and are summarised below.

In the study by Sethi et al. on 300 urban families [46], their survey team visited each family and interviewed head of the family or the house wife, and a comprehensive questionnaire was administered. Based upon the information obtained, the numbers of individuals with psychiatric manifestations were identified. These patients were then clinically evaluated by a fully trained psychiatrist. Similar methodology was also adapted by Sethi et al. [46], which was conducted on 500 rural families [47]. In both the studies though all age groups were covered, for the purpose of this analysis the data of 0–20 year age group population was taken into account.

Elnagar et al. [48] carried out their study in three stages. In the first stage of case finding, a house to house survey of all the 184 families was done. In the second stage detailed case histories of suspected cases were taken. The third stage of the survey was confirmation from a clinical psychiatrist. They defined a case of mental illness as per the definition by WHO expert committee on mental health convened in 1960.

Nandi et al. [18,49,50] prepared a questionnaire in local language after consultation with other psychiatrists. The case record schedule prepared by them gave all relevant information regarding the case detected through the questionnaire. They defined a ‘case’ as per WHO technical report series no. 185 (1960) with certain modifications.

Singh et al. [19] in their study adopted the following methodology. In first stage they initially interviewed the parents, usually mothers on present and past histories of a mental illness, schooling and early life details of their children, followed by physical and psychiatric examination of every child individually. They defined the case as per WHO technical report series no. 185 (1960) with certain modifications.

Thacore et al. [51] in their study first interviewed the head of the family, the housewife or a responsible member of the family. Individuals suspected of suffering from mental illness were then examined in detail. An individual was considered a ‘psychiatric case’ if he showed disturbance in mental functioning which was clinically recognizable. They made the diagnosis as per the DSM II. In the study by Shah et al. [52] the detection of the index case was done by the senior post graduate students using a 58 question symptom checklist and clinical interview was done by the consultant to make the diagnosis.

Mehta et al. [53] conducted the study in eleven villages. For the detection of psychiatrically disordered individuals, the ‘symptoms in others’ questionnaire part of the Indian Psychiatric Survey Schedule [54] was used. Individuals found to have any of the symptoms, mentioned in the questionnaire were examined by a psychiatrist to confirm as to whether they had any psychiatric disorders. Sachdeva et al. [55] used similar methodology like that of Mehta et al. Singh et al. [56] used a reporting questionnaire consisting of 11 items as a screening instrument. They applied this questionnaire to parents of 348 children aged 5–15 years from 200 families. After completion of screening, the identified children were clinically assessed in detail. Vergese and Beig [57] selected the household for their study by a three stage random sampling method. Mental health of each member of the household was assessed by means of a validated mental health item sheet. If any member showed any suggestion of psychiatric disturbance, he or she was evaluated in detail. They also administered Seguin Form Board test and made the diagnosis as per the ICD-8 (1964). Premrajan et al. [58] in their study used a pre-tested questionnaire, which was a modified form of the Indian Psychiatric Survey Schedule [54]. The parents of the children were interviewed and if found to have any psychiatric symptoms, were subsequently evaluated clinically by a psychiatrist. The diagnosis was made as per ICD-9. Gaur et al. [59] selected 400 children from rural and 400 children from urban area using systematic random sampling. All children in the selected sample were subjected to a screening for psychiatric symptoms by interviewing the parents or key informant of the children using the Hindi version of Childhood Psychopathology Measurement Schedule [60]. The children screened positive for psychiatric morbidity (score ≥ 10) were subjected to diagnostic assessment using diagnostic interview schedule for children. The diagnosis was made as per ICD-10 criteria.

The ICMR study was carried out in Bangalore [61]. The sample was selected by stratified multistage sampling from middle class urban, urban slum and rural areas. They used screening checklist (SCL), child behaviour checklist (CBCL), additional module, children’s behaviour questionnaire (CBQ), felt treatment need (FTN), diagnostic interview for children, structured interview schedule, parent interview schedule, Vineland social maturity scale (VSMS), Binet Kamath test, SLD battery, and children’s global assessment scale. In the first (screening) stage all children aged 0–16 yr were screened using age appropriate screening instruments. Children selected as positive in the first stage were taken for a detailed evaluation. ICD-10 DCR diagnoses were assigned after clinical interviews. This study is relatively methodologically sound than other epidemiological study from India. The sample size is quite adequate to ensure that cases of psychiatric disorders with an expected prevalence rate of 1% or higher were not missed out. Multi-stage sampling procedure has been used rather than purposive or convenience sampling. This can help in estimating more accurate prevalence rates. Separate prevalence rate for middle class urban areas and urban slum areas have been estimated in this study, as there may be different determinants of mental health in these two settings. This study also used a number of instruments and studied more number of psychiatric disorders than other epidemiological studies from India.

### School based studies

Apart from the community based epidemiological studies many investigators studied the childhood psychiatric disorders in the school. Out of a total of 25 studies, 7 studies were done on specific disorders [30-36] and in nine studies children were not interviewed [20,62-69]; so they were excluded from the analysis. Studies where the respective journals were not available were also not included in analysis [70,71]. Thus a total of seven school based studies were included in the analysis.

The school based studies have been shown in the Table 2.

**Table 2:** School based studies included in analysis

**Sl no. P.I.**	**year**	**State/UT**	**Setting**	**Age group**	**N**	**Prev. (%)**
1. Jiloha [72]	1981	Haryana	rural	5-12	715	20.7
Used questionnaire (t) and reporting questionnaire for children (p), clinical interview. Diagnosis was made as per ICD-9.						
2. Devsigamani [22]	1990	TN	urban	8-12	755	33.7
Rutter B scale was used as a screening instrument and diagnosis was made as per ICD- 9 after clinical interview.						
3. Mehta [74]	1997	WB	rural	8-10	8-10	460
Rutter B scale (Hindi adaptation), Malin’s test of verbal intelligence, VSMS, Gessell’s drawing test were used. Diagnosis was made as per DSM-III-R.						
4. Banerjee [75]	1997	WB	rural	8-10	460	33.33
Rutter’s B scale was used as screening instrument and ICD-9 was used to make the diagnosis						
5. Gupta [73]	2001	Punjab	urban	9-11	957	45.6
Rutter’s B scale was used as screening instrument and ICD-10 was used to make the diagnosis						
6. Malhotra [76]	2002	Chandigarh	urban	4-11	963	6.33
Rutter B scale, pre-school behaviour checklist, Childhood Psychopathology Measurement Schedule was used. Diagnosis was made as per ICD-10.						
7. Bansal [77]	2011	Punjab	urban	10-15	982	20.2
Childhood Psychopathology Measurement Schedule was used and Diagnosis was made as per ICD-10.						

### Methodology of included school based studies

The study done by Jiloha and Murthy [72] drew sample from 4 primary schools. During Stage 1 teachers and parents identified children with emotional problems and during Stage 2, identified children were assessed with psychological tests. They applied questionnaire to teachers and parents and clinical interview was also done. The diagnosis was made as per the ICD-9. Gupta et al. [73] conducted their study on 1000 school children aged 9–11 years. In the first stage, screening instrument Rutter B scale was given to the teachers to rate the child’s behaviour in school. Children with a score of ≥ 9 were considered as deviant and included in the second stage involving a semi structured interview and diagnosis was made as per ICD-10 guidelines. An equal number of sex matched children selected by simple random sampling who scored < 9 were also clinically interviewed along with parents [73].

Mehta et al. [74] conducted the study in 4 schools. The teachers identified the children scoring above 9 on Hindi adaptation of Rutter’s Behaviour Questionnaire: Scale B. Detailed assessment of children scoring above 9 on Rutter’s questionnaire was done. Malin’s tests of verbal intelligence, Vineland Social Maturity Scale, Gessell’s drawing test were used for assessment of IQ. Diagnosis was made as per DSM-III-R after detailed assessment. This study has got some methodological advantages. This was done in four schools with a good sample size of 855 children. The school teachers were assessed regarding knowledge, attitude, practice on the presence and recognition of mental health problems. Based on the results of the survey, lectures and discussion on mental disorder for orientation of teachers was done. The school teachers were then introduced to the screening device, “Rutter’s Children’s Behaviour Questionnaire” which was translated and validated in the local language and after that each child was rated by the teachers. Psychometric tests have also been done along with clinical interviews.

Banerjee [75] studied psychiatric morbidity in 460 rural primary school children of aged 8–10 years. In the first stage all of these children were screened by Rutter’s B Scale. In his study, he kept the cut off score of 17/18 on the basis of a previously conducted validity study of this scale in rural school children. The children above the cut-off score (n = 125) were clinically examined and parental interview were also carried out. The diagnosis was made as per ICD-9.

Deivasigamani [22] studied 755 children aged 8–12 years from six schools for his study. In the first stage a screening instrument Rutter B scale was used to screen all the children. The children scoring ≥ 9 were selected. Equal numbers of children scoring < 9 were picked up from the remaining children randomly. The second stage of the study involved parental interview and clinical evaluation. Diagnosis was made as per ICD- 9 after clinical interview.

Malhotra et al. [76] selected 963 children aged 4–11 year. The sampling procedure involved random selection of schools stratified on the basis of type of school and gender of the student; and selection of children from the selected school, stratified according to age categories. The children were categorised in 4 age groups with 200 children in each age group, which would provide sufficient numbers of disordered children in each group. The study was conducted in three stages, which is a strength of this study. The stage 1 comprised the teacher’s assessment of the child’s behaviour using Rutter’s B scale (Hindi version). For children 4–5 year old, the pre-school behaviour checklist was chosen. All the children scoring ≥ 9 were considered positive at stage 1 and were assessed in detail (stage-3). In the stage-2, the parents of all the children seen at stage 1 were contacted at home. The mother’s were interviewed on Childhood Psychopathology Measurement Schedule (CPMS) and other instruments to measure psychiatric symptoms, temperament, and parental handling and life events (stage-2). The Childhood Psychopathology Measurement Schedule is an Indian adaptation of Achenbach’s Childhood behaviour Checklist. A score of ≥ 10 on CPMS indicated the possibility of psychiatric disorder and these children were clinically assessed in detail (stage-3). The evaluation was done in detail by the psychiatrist. IQ testing was done using MISIC, VSMS and Gessel’s drawing test. Diagnosis was made as per ICD-10. Different prevalence rates were also been reported based on informants i.e. teacher’s estimate was 10.17%, parents’ estimate was 7.48% and overall prevalence was found to be 6.33%.

The study by Bansal and Barman [77] was done on children aged 10–15 years using CPMS. The children who score ≥ 10 were assessed further and interviewed clinically and were diagnosed according to ICD-10 criteria.

### Estimation of prevalence rates

It was recognized that the studies were heterogeneous both in terms of the criteria used to define the cases and disorders studied. Because of the above reason we calculated the weighted mean of overall prevalence of psychiatric disorders. The weighted mean was calculated by dividing the sum of the product of prevalence (as percentage) and population studied by the sum of the total population.

The weighted mean average prevalence rate of psychiatric disorder was found to be 6.46% (95% confidence interval 6.08% - 6.88%) for the community based studies. For the school based studies the prevalence was found to be 23.33% (95% confidence interval 22.25% - 24.45%).

## Discussion

In this analysis the weighted mean average prevalence rate of psychiatric disorder was found to be 6.46% (95% confidence interval 6.08% - 6.88%) in the community. This figure is similar to the epidemiological studies conducted in UK and Puerto Rico which show the prevalence to be 6.8% [78] and 6.9% [17] respectively. However, the prevalence was much higher in some other countries like Germany 20.7% [79], 14.5% [80] and Switzerland 22.5% [81].

There is a wide variation in the prevalence rate of child and adolescent psychiatric disorders. This could be due to various reasons. The most important among these is definition of a ‘case’. Various studies carried out in India and abroad used different criteria to define a ‘case’. The main problem is the absence of a gold standard that could be used as a uniformly acceptable criterion. In an older study, a statistical definition was used, where a symptom was considered abnormal if it occurred in 10% or less of the children studied [82]. As per these nosological systems, a ‘case’ is defined as an individual who has a disorder which causes significant distress and/or dysfunction to self or others. The level of distress or dysfunction to become ‘significant’ varies from culture to culture and individual to individual. This is because the tolerance to child behaviour differs across cultures [83-85]. So the instruments that measure the informant’s perception of behaviour are liable to be fallacious, identifying the children as abnormal as per the values bestowed upon them by their culture. Selection of culturally appropriate tools in India is an important issue in epidemiological studies. Research exploring the applicability of Western tools with or without slight modification is a good milestone of development. An important development in this area is the development of The Childhood Psychopathology Measurement Schedule, which is an indigenous screening tool and has been standardized in the Indian children [60].

The prevalence rate in childhood psychiatric disorders also depends on the methodology i.e. whether children were interviewed or not and the informants interviewed as there is low agreement among informants i.e. - parents, teachers, peers; and with the clinicians and different prevalence rates have been reported across informants [22,60,86]. Though teachers are good informants they may not be aware of the problems occurring in home (e.g. enuresis) or less likely to be reported the internalising or emotional problems. Thus, prevalence studies without interviewing the child are likely to yield inaccurate estimates and were not included in this analysis. Though one limitation may be possibility of excluding studies that used standardized and culturally validated instruments [45]. Sample size is another source of variation in calculating prevalence rates. Sample sizes ranged from 273 to 2064 in community studies [58,61] and 460 to 982 in school based studies included in this analysis [75-77]. To detect cases of psychiatric disorders with an expected prevalence rate of 1 per cent or higher, so that no case are missed out, a sample size of at least 2000 is required [61]. The relatively small sample size and inclusion of a specific group of children i.e. either preschool/school going/ adolescence is a major limitation. From a developmental perspective, it is difficult to report separate prevalence rates for pre-school, childhood and adolescence. Large samples, spanning all three groups, would provide a better understanding of the epidemiological distribution of childhood psychiatric disorders [87]. The use of purposive or convenience sampling in many studies resulted in decreased precision in prevalence estimates. Some studies used the multi-stage designs to estimate more accurate prevalence rates [61,76].

The community and school based studies were analysed separately. This was done because the two settings represent different psychiatric morbidities. Children with intellectual disabilities, brain damage and consequent neuropsychiatric morbidities, and epilepsy which are more common in LAMIC like India are more likely to be represented in the community sample than the school based sample [88]. The children with learning or emotional problems are also more likely to drop out from school [89]. The both samples are analysed separately to reduce the heterogeneity. The weighted mean average prevalence rate of childhood psychiatric disorder in school was found to be 23.33% (95% confidence interval 22.25% - 24.45%) in our analysis. Other studies show the prevalence rates ranging from 16.2% in Germany [90] and 30% in New Zealand [91]. The prevalence rate in the school obtained by this analysis was found to be different than that of community.

The wide variation between community based prevalence and school based prevalence rate may be due to many factors. As there was limited number of community based studies exclusively done on child and adolescent population (from 0–19 years), data from studies done on all age groups were taken for this analysis for the community based studies. However, studies done in the schools cater only to the child and adolescent population within 5–19 years age group. As psychiatric morbidity in 0–5 years age group would be very low it may have brought down the overall prevalence rate in the in the community samples. Moreover, the school based studies included in this analysis were carried out after 1990 except only one study [72] where as out of sixteen community based studies which were included in this analysis, twelve were before 1990 and only four studies were after 1990. So there were differences in the diagnostic systems used i.e. the older studies used the earlier version of diagnostic systems such as ICD-9 or DSM- II& DSM III where the diagnostic criteria were less clear. Further there may also be changing trend in child and adolescent psychiatric disorders over time. For analysis of the school based studies only seven studies could be included and 5687 children were studied. In the community based studies sixteen studies could be included with a total study population of 14594. More studies with larger sample size and would give more accurate results in epidemiological studies.

As per the UNICEF data in 2008, number of persons below 18 year was around 447 million in India [92]. The 6.46% prevalence rate means, out of 447 million children and adolescent population, 29 million suffer from one or other form of psychiatric illness at a given time. It is difficult for a country like India to handle such a huge problem with meagre mental health resource coupled with very slow development in child and adolescent psychiatry [93]. Though the WHO recommended in 1977 that every country throughout the world should have a National Plan for Child Mental Health, India does not have a child and adolescent mental health programme [9]. So there is an urgent need to develop mental health resources for children and adolescent in India.

## Conclusion

This is the first meta-analysis determining the epidemiology of child and adolescent psychiatric disorders in India. It has been found that the reporting systems of psychiatric disorders in children are inadequate. Case definition and recognition of disorders varies across states or has variable interpretations; and above all the cultural components of what constitutes a disorder remain an important issue. Though it has been suggested that meta-analysis should be carried out once in ten years, there is no meta-analytic study for childhood and adolescent psychiatric disorder so far. This piece of work is the first attempt in this area in India representing a prevalence of 6.4% in community samples and 23.33% in school samples. The need for methodologically sound epidemiological studies for children and adolescent in India is highlighted.

## Competing interests

The authors declare that they have no competing interests.

## Authors’ contributions

(SM): Conception and design of the paper, editing and revising the manuscript critically for important intellectual content, final approval of the version to be published. (BNP): Planning of the paper, reviewed and collected the literature, initial drafting of the manuscript and final approval of the version to be published.

## Authors’ information

SM is currently Professor and Head of the Department of Psychiatry at Postgraduate Institute of Medical Education and Research, Chandigarh, India. She is the life president of Indian Association for Child and Adolescent Mental Health and currently the president elect of the Asian Society for Child and Adolescent Psychiatry & Allied Professions.

BNP has done his MD (Psychiatry) from All India Institute of Medical Sciences, New Delhi and recently completed his Senior Residency from the Department of Psychiatry at Postgraduate Institute of Medical Education and Research, Chandigarh, India.
