# Erratum to: Prevalence of child and adolescent psychiatric disorders in India: a systematic review and meta-analysis

**DOI:** 10.1186/s13034-017-0175-2

**Published:** 2017-07-12

**Authors:** Savita Malhotra, Bichitra Nanda Patra

**Affiliations:** Department of Psychiatry, Postgraduate Institute of Medical Education & Research, Chandigarh, 160012 India

## Erratum to: Child and Adolescent Psychiatry and Mental Health 2014, 8:22 DOI 10.1186/1753-2000-8-22

After publication of the article [1] it was brought to our attention that some of the data from Table 2 has been inputted incorrectly. In Table 2, the first line of point 3 should be read as

**Table:** 

Mehta [74]	1991	Haryana	rural	6–12	855	8.9
